# Identification of the Major Sex Pheromone Component of the Click Beetle *Agriotes ferrugineipennis*

**DOI:** 10.1007/s10886-022-01367-1

**Published:** 2022-07-27

**Authors:** Kendal Singleton, Regine Gries, Willem G. van Herk, Santosh K. Alamsetti, Emily Lemke, Kathleen Furtado, Gerhard Gries

**Affiliations:** 1 Department of Biological Sciences, Simon Fraser University, Burnaby, British Columbia V5A 1S6 Canada; 2 Agassiz Research and Development Centre, Agriculture and Agri-Food Canada, British Columbia V0M 1A0 Agassiz, Canada

**Keywords:** Click beetles, Elateridae, *Agriotes ferrugineipennis*, Sex pheromone, Identification, 7-methyloctyl 7-methyloctanoate

## Abstract

Synthetic sex pheromone lures are useful tools to monitor and control populations of adult click beetles (Coleoptera: Elateridae). However, sex pheromones for *Agriotes* click beetle species native to North America have yet to be identified. Here we report the identification and field testing of the sex pheromone of *Agriotes ferrugineipennis.* Headspace volatiles from female beetles were collected on Porapak Q, and aliquots of Porapak extract were analyzed by gas chromatographic-electroantennographic detection (GC-EAD) and GC-mass spectrometry. 7-Methyloctyl 7-methyloctanoate (7Me7Me) emitted by females was more abundant and elicited much stronger responses from male antennae than the aldehydes octanal and nonanal and the ketone 6,10,14-trimethyl-2-pentadecanone. In a field experiment, captures of *A. ferrugineipennis* males in traps baited with candidate pheromone components exceeded those of unbaited control traps, on average by nearly 1,200 times. Neither the ketone nor the aldehydes as lure constituents appeared to alter captures of males in 7Me7Me-baited traps. We conclude that 7Me7Me is the major, and possibly the only, sex attractant pheromone component of female *A. ferrugineipennis.*

## Introduction

Wireworms, the larvae of click beetles (Coleoptera: Elateridae), are ubiquitous soil-dwelling pests (Poggi et al. 2021), feeding on many agricultural crops (Traugott et al. 2008, 2015). In recent years, populations of pestiferous click beetles have regained economic importance, possibly due to the deregistration of insecticides (van Herk et al. 2021a), insecticidal residues leaching out of arable land, and agricultural practices such as no-tillage farming and ‘land set-aside’ schemes (Jedlička and Frouz 2007; Traugott et al. 2015; Vernon and van Herk 2022). Tillage alters the soil microclimate, destroys beetle eggs and larvae, and brings them to the soil surface where they desiccate or fall prey (Lees 1943; Saussure et al. 2015).

Synthetic sex pheromone lures of click beetles are useful tools to (*i*) monitor population dynamics of adult beetles, (*ii*) delineate the geographic distribution of species, (*iii*) help predict crop damage, (*iv*) time insecticidal control measures, (*v*) detect the presence and track the spread of invasive species, and (*vi*) surveil the displacement of native species by invasive species (e.g., Kudryavtsev et al. 1993; Tolasch et al. 2007; Musa et al. 2013; Traugott et al. 2015; Furlan et al. 2020; van Herk et al. 2021b). Also, synthetic sex pheromones are increasingly considered for control of adult beetle populations through mass trapping, mating disruption, and attract & kill tactics (e.g., Reddy and Tangtrakulwanich 2014; Vernon et al. 2014; Kabaluk et al. 2015; Vernon and van Herk 2022).

To date, sex pheromones are known for only eight elaterid species native to North America, including *Melanotus communis* (Williams et al. 2019), *Cardiophororus tenebrosus* and *C. edwardsi* (Serrano et al. 2018), *Limonius canus* and *L. californicus* (Gries et al. 2021; van Herk et al. 2021d), *Selatosomus aeripennis destructor* (Gries et al. 2022), *Idolus californicus* (Serrano et al. 2022), and *Parallelostethus attenuatus* (Millar et al. 2022). Sex pheromones are not yet known for any North American-native *Agriotes* click beetles such as *Agriotes ferrugineipennis.*

The genus *Agriotes* is of particular agricultural importance, with about 20 of the > 200 described species (Becker 1956) being significant agricultural pests in Europe (Tóth 2013;  Ritter and Richter 2013; Traugott et al. 2015), North America, and Asia (Vernon and van Herk 2022). In the UK and northern Europe, *A. lineatus, A. obscurus,* and *A. sputator* are the predominant pest wireworm species in agricultural land (Parker and Howard 2001). These three species established in Canada in the 1800s, with *A. lineatus* and *A. obscurus* now being important pests of field crops in southern British Columbia (BC) (Wilkinson 1963; van Herk et al. 2021c), and all three being pests in Eastern Canada since the 1800s (Eidt 1953; Vernon and van Herk 2022).

Adult beetles of *A. ferrugineipennis* are medium-sized (9–12 mm) with a distinct reddish hue on their antennae and legs, and pronounced hind angles of the pronotum (Becker 1956). The beetles occur throughout BC, Alberta, Washington, California, Idaho, Oregon, Nevada, and Utah (Becker 1956; Wilkinson 1963; van Herk et al. 2021b). Although found in agricultural land, the pest status of *A. ferrugineipennis* is unclear (Glen 1944; Wilkinson 1963).

Males of *A. ferrugineipennis* reportedly respond to abdominal extracts of conspecific females but the compound(s) mediating the attraction responses of males remained unknown (Lilly and McGinnis 1965). Here we report the identification and field testing of the major sex pheromone component of female *A. ferrugineipennis.*

## Material and Methods

### Field Collection of Beetles

In May and June 2020, click beetles were collected in Pemberton, BC, at potato fields with historically high *A. ferrugineipennis* populations. Crops were rotated every three years, and at the time of collection, the field was covered with grass from which beetles were collected with sweep nets. Captured beetles were separated by species and their sex was determined by careful extrusion of their genitalia. Due to a paucity of beetles, a group of only three females and 30 males were collected. These groups were maintained in separate plastic cups (140 mL; Fisher Scientific, Ottawa, ON, CA) with perforated lids to facilitate air exchange. Cups contained fresh grass for both moisture and walk-on substrate for beetles, and small pieces (2 × 2 cm) of apple for food. All cups were kept at a low temperature (~ 4 °C) to extend the beetles’ longevity. Prior to collecting the beetles’ headspace volatiles, cups were warmed to room temperature and the grass was replaced with a moist Kim wipe. Apple pieces were replaced once a week or when they had become soft and moldy, and Kim wipes (Fisher Scientific, Ottawa, ON, CA) were remoistened as needed. To reduce beetle mortality, cups were replaced every two weeks or when a beetle had died.

### Collection of Headspace Volatiles

Headspace volatiles of beetles were collected following a protocol previously detailed (Gries et al. 2021). Briefly, the three females and the 11 males of *A. ferrugineipennis* we had available for volatile captures were placed into separate Pyrex® glass chambers (8 cm high × 8 cm diameter), each fitted with a moist cotton wick (Richmond Dental, Charlotte, NC, USA) as a source of water and walk-on substrate. A mechanical pump (Neptune Dyna-pump, Model 2 Dover, NJ, USA) drew charcoal-filtered air at a flow of 0.5 L · min^−1^ for 24 h through the chamber and through a glass column (6 mm outer diameter × 150 mm) containing 200 mg of manufacturer-preconditioned Porapak-Q™ adsorbent (50–80 mesh; Waters Associates, Milford, MA, USA). Porapak Q volatile traps was desorbed with pentane/ether (2 mL, 50:50) and concentrated to 100 µL for analyses.

### Gas Chromatography with Electroantennographic Detection (GC-EAD) Analyses

Aliquots of the Porapak Q extract of female beetles, and of synthetic standards, were analyzed by GC-EAD, with equipment and procedures previously detailed (Gries et al. 2002). Briefly, the GC-EAD setup employed a Hewlett-Packard 5890 gas chromatograph (GC) fitted with one of four GC columns (DB-5, DB-210, DB-23, FFAP; all 30 m × 0.32 mm ID; film thickness 0.25 µm; Agilent J & W column, Agilent Technologies Inc., Santa Clara, CA, USA). Helium served as the carrier gas (35 cm · s^−1^) with the following temperature programs: 50 °C for 1 min, then 20 °C · min^−1^ to 220 °C (DB-210, DB-23) or 280 °C (DB-5); 100 °C for 1 min, then 20 °C · min^−1^ to 180 °C (held for 15 min) (FFAP). The injector port and flame ionization detector (FID) were set to 260 °C and 280 °C, respectively. For each GC-EAD recording, an antenna was carefully dislodged from a male’s head and suspended between two glass capillary electrodes (1.0 × 0.58 × 100 mm; A-M Systems, Carlsborg, WA, USA) prepared to accommodate the antenna and filled with a saline solution (Staddon and Everton 1980). Antennal responses to compounds in the column effluvium – that was directly released into a stream of medical air (250 mL/min flow) continuously passing over the electrode-suspended antenna – were amplified with a custom-built amplifier and recorded on an HP 3392A integrator. The voltage of antennal responses was derived from correlations between peak height and integrator attenuation, as tabulated in the recorder manual. Stable GC retention times made it possible to direct the entire column effluent, in sequence, to the FID and the EAD, thus allowing us to align EAD responses to FID peaks while increasing the probability of detecting minor sex attractant pheromone components. Because only eight males were available for analyses, and not every antennal preparation was functional, just one or two usable GC-EAD recordings could be obtained on each of the four GC columns (see above).

### GC-Mass Spectrometry and NMR Spectroscopy

Headspace volatiles that elicited antennal responses were deemed candidate pheromone components (CPCs) and were analyzed by GC–MS, using both a Varian Saturn 2000 Ion Trap GC–MS and a 5977A Series MSD (both Agilent Technologies Inc., Santa Clara, CA, USA) coupled to a 7890B GC. Both instruments were operated in full-scan electron ionization mode and fitted with a DB-5MS column (30 m × 0.25 mm ID; Agilent J&W GC), using helium as the carrier gas (35 cm · s^−1^). The injector port and ion trap were set at 250 °C and 200 °C, respectively, and the temperature program was as follows: 50 °C for 5 min, 10 °C · min^−1^ to 280 °C (held for 10 min). To identify CPCs in Porapak-Q headspace volatile extract, their retention indices (Van den Dool and Kratz 1963) and mass spectra were compared with those of authentic standards that were purchased or synthesized. The ^1^H-NMR spectra of a synthetic candidate pheromone component and of two model compounds, were recorded on a Bruker 500 MHz spectrometer using CDCl_3_ as solvent. Signal positions (δ) are given in ppm from tetramethylsilane (δ 0) and were measured relative to the signal solvent (^1^H NMR: CDCl_3_: δ 7.26). Coupling constants (*J*) are given in Hertz (Hz) and are reported to the nearest 0.1 Hz. ^1^H NMR spectral data are tabulated in the order: multiplicity (s, singlet; d, doublet; t, triplet; q, quartet; m, multiplet; br., broad), coupling constants, number of protons.

### Chemicals

#### Synthesis of 7-methyloctyl 7-methyloctanoate (7Me7Me), 6-methyloctyl 6-methyloctanoate (6Me6Me), and 5-methyloctyl 5-methyloctanoate (5Me5Me)

All synthetic acid intermediates (7-methyl octanoic acid, 6-methyl octanoic acid, 5-methyl octanoic acid) were purchased (Toronto Research Chemicals; North York, ON, CA) and the corresponding alcohols were produced by reduction of these acids with lithium aluminum hydride (LiAlH_4_) (Jones and Fleming 1997). Esters were obtained following a well-established method (Neises and Steglich 1978), using dicyclohexylcarbodiimide (DCC) and 4-dimethylaminopyridine (DMAP) as coupling reagent and catalyst, respectively, with yields ranging between 68–73%. ^1^H NMR data of 7Me7Me were consistent with those previously reported (Tolasch et al. 2007) and the mass spectrum is shown in Fig. 3. ^1^H NMR and GC–MS data of 6Me6Me: ^1^H NMR (500 MHz, CDCl_3_): δ 4.06 (t, *J* = 6.7 Hz, 2H), 2.30 (t, *J* = 7.5 Hz, 2H), 1.65–1.51 (m, 5H), 1.32 – 1.21 (m, 8H), 1.11 (dq, *J* = 12.7, 6.3, 5.8 Hz, 3H), 0.9 – 0.82 (m, 16H); the mass spectrum of 6Me6Me is shown in Fig. 3. ^1^H NMR and GC–MS data of 5Me5Me: ^1^H NMR (500 MHz, CDCl_3_): δ 4.06 (t, *J* = 6.7 Hz, 2H), 2.30 (t, *J* = 7.5 Hz, 2H), 1.65–1.51 (m, 5H), 1.32 – 1.21 (m, 8H), 1.11 (dq, *J* = 12.7, 6.3, 5.8 Hz, 3H), 0.9 – 0.82 (m, 16H); the mass spectrum of 5Me5Me is shown in Fig. 3.

All three esters were purified for NMR analyses by HPLC (Waters Corporation, Milford, MA, USA: 600 Controller, 2487 Dual Absorbance Detector, Delta 600 pump) fitted with a Spursil RP C18 column (3 µm, 250 mm × 4.6 mm; Dikma Technologies Inc., Lake Forest, CA; USA) eluted with an isocratic flow (1 ml/min) of acetonitrile.

6,10,14-Trimethyl-2-pentadecanone was available from a previous project (Sasaerila et al. 2003), and octanal and nonanal were purchased (Sigma Aldrich, St Louis, MO, USA). The chemical purity of field-tested octanal, nonanal, 6,10,14-trimethyl-2-pentadecanone and 7Me7Me was 99%, 95%, 99% and 96%, respectively.

### Field Trapping Experiment

**Fig. 1:**
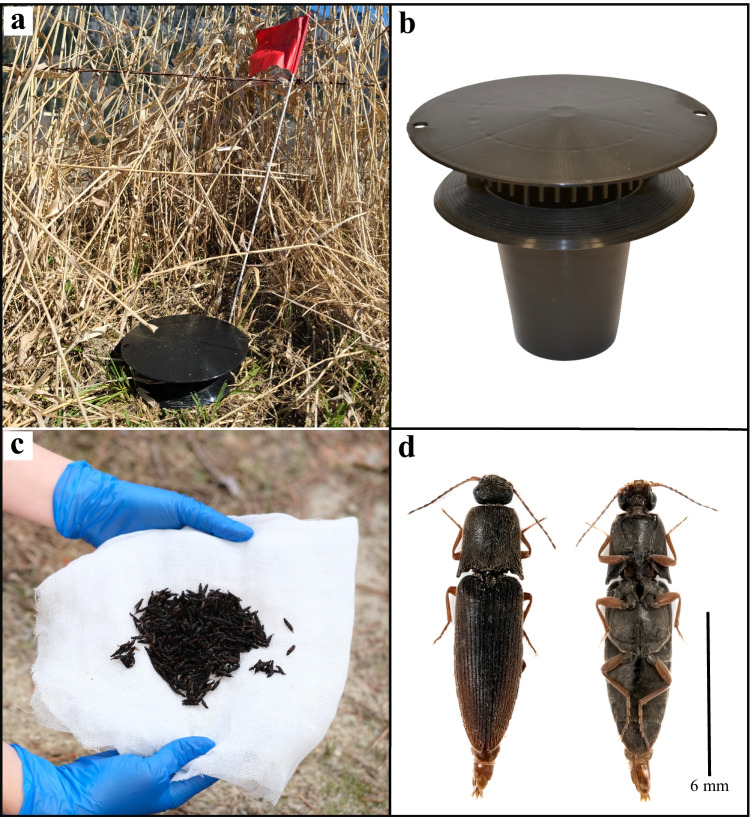
(**a**, **b**) Photographs of a Vernon Pitfall Trap placed in the field for testing candidate pheromone components (**a**) and in unobstructed view to reveal the trap bottom and lid (**b**); (**c**) a representative sample of *Agriotes ferrugineipennis* males captured in a single pheromone-baited trap over the course of seven days in Pemberton, British Columbia; (**d**) dorsal and ventral views of a single *A. ferrugineipennis* male

The experiment was run in two adjacent fields (each 5.83 ha, 4.59 ha) near Pemberton, BC (50.429236, -122.907198) from which beetles had been collected for pheromone identification. The experiment followed a general protocol previously detailed (Gries et al. 2021), using a complete randomized block design with eight replicates situated in each field. Four additional replicates were placed in a grassy berm along a driveway leading up to one of the fields. Vernon pitfall traps (van Herk et al. 2018; available from Intko Supply Ltd., Chilliwack, BC, CA) were placed at ground level along the field’s edge (Fig. 1a), with 10-m and 20-m spacing between treatments and replicates, respectively. Traps were baited with synthetic CPCs (see below) dissolved in hexane of which 45-μL aliquots were pipetted onto 100% cotton pellets (size #0; Richmond Dental, Charlotte, NC, USA). The cotton pellets were placed inside of 1-mL LDPE containers (diameter: 8 mm, height: 32 mm; wall thickness: 0.98 mm; product number: 00730; Kartell Labware, Noviglio, IT) which were open and suspended from the roof of traps. Each experimental replicate (*N* = 16 during weeks 1–3; *N* = 20 during weeks 4–7) consisted of five treatments: (1) an unbaited control; (2) 7-methyloctyl 7-methyloctanoate (7Me7Me) (10 mg); (3) a ternary blend of 7Me7Me (10 mg), octanal (1 mg) and nonanal (1 mg); (4) a binary blend of 7Me7Me (10 mg) and 6,10,14-trimethyl-2-pentadecanone (1 mg); and (5) a quaternary blend of 7Me7Me (10 mg), octanal (1 mg), nonanal (1 mg), and 6,10,14-trimethyl-2-pentadecanone (1 mg). As only 7Me7Me was female-specific (see Results) and thus deemed to be the major candidate pheromone component, it was field-tested at a dose tenfold higher than that of the other EAD-active components even though the two aldehydes were as abundant as 7Me7Me in the headspace of females. The first 16 replicates of the experiment were installed on 12 April 2021, and the remaining four replicates on 3 May 2021. The experiment was terminated on 31 May 2021. Traps were checked and captured beetles were collected every seven days. Total counts of captured beetles were recorded and beetles in subsamples were identified to species and sex.

### Identification of Captured Beetles

Click beetles were identified to species level using taxonomic keys (Becker 1956). Specimens that were taxonomically ambiguous were identified solely based on genitalia characteristics, which are distinct for male *A. ferrugineipennis* (Becker 1956). Specimens with missing genitalia and otherwise badly damaged were excluded from analysis. A total of 88 (of 151) beetles captured in unbaited control traps and subsamples of > 480 beetles captured in traps assigned to each of the four pheromone treatments (see above) were identified to species. Each of these subsamples consisted of five samples (up to 20 beetles each) taken from every collection week of the 7-week study (Table 1). Voucher specimens are retained at the Agassiz Research and Development Centre (Agassiz, BC, CA).

**Table 1:** Mean (SEM) proportions, and estimated total numbers, of *Agriotes ferrugineipennis* males (relative to all click beetles captured) collected in traps left unbaited (control) and in traps baited with 7-methyloctyl 7-methyloctanoate (7Me7Me) alone and in combinations with the aldehydes octanal and nonanal, the ketone 6,10,14-trimethyl-2-pentadecanone, and both the aldehydes and the ketone (Pemberton, British Columbia, 12 April to 31 May 2021; *N* = number of replicates)

Collection date	*N*	Stimuli tested				
		Unbaited	7Me7Me	7Me7Me + aldehydes	7Me7Me + ketone	7Me7Me + aldehydes + ketone
Mean (SEM) proportion of male *A. ferrugineipennis*						
19 April	5	0 (0)	0.03 (0.03)	0.50 (0.00)	0.52 (0.27)	0.13 (0.13)
26 April	5	0 (0)	0.38 (0.19)	0.38 (0.18)	0.38 (0.17)	0.42 (0.14)
03 May	5	0.21 (0.15)	0.87 (0.07)	0.96 (0.02)	0.99 (0.01)	0.99 (0.01)
10 May	5	0.18 (0.12)	0.94 (0.04)	0.97 (0.02)	0.98 (0.01)	0.97 (0.03)
17 May	5	0 (0)	0.99 (0.01)	0.97 (0.03)	0.98 (0.02)	0.99 (0.01)
24 May	5	0 (0)	0.93 (0.06)	0.91 (0.05)	0.88 (0.07)	0.89 (0.06)
31 May	5	0 (0)	0.83 (0.13)	0.89 (0.10)	0.78 (0.20)	0.81 (0.12)
total beetles collected		151	8246	6694	7708	8384
total beetles identified		88	506	480	488	490
Mean (SEM) number of male *A. ferrugineipennis*						
19 April	16	0 (0)	0.3 (0.1)	1.8 (0.7)	9.9 (3.6)	1.1 (0.4)
26 April	16	0 (0)	10.6 (4.1)	7.5 (2.7)	16.4 (5.6)	15.4 (6.0)
03 May	16	0.1 (0.1)	42.6 (19.6)	38.6 (18.6)	68.6 (25.2)	61.2 (29.4)
10 May	20	0.2 (0.1)	109.5 (21.9)	90.8 (16.9)	105.9 (16.3)	122.1 (19.7)
17 May	20	0 (0)	180.6 (25.0)	145.2 (20.4)	139.0 (17.2)	165.5 (19.2)
24 May	20	0 (0)	30.6 (6.3)	26.2 (5.1)	19.5 (4.4)	28.1 (4.5)
31 May	20	0 (0)	8.9 (3.2)	11.1 (3.4)	6.4 (2.0)	8.2 (1.6)
Sum		0.3 (0.1)	372.2 (59.5)	311.5 (47.9)	346.6 (48.8)	386.0 (54.5)

### Statistical Analyses of Data

To determine whether the proportion of trap-captured *A. ferrugineipennis* males varied with treatment and collection week, beetle subsamples were selected randomly for each treatment from five replicates per collection week, as mentioned above. Proportions were compared using generalized linear models with a binomial distribution and a logit link function (Proc GENMOD, SAS 9.2, SAS Institute, Cary, NC, USA), and mean proportions were calculated per treatment and collection week (Table 1). These mean proportions were then used to calculate the number of *A. ferrugineipennis* males collected per trap per week, and the interpolated number of beetles was summed over the 7-week collection period to calculate the total number of *A. ferrugineipennis* males collected per trap. Differences between treatments were analyzed using total counts with generalized linear models fitted with a negative binomial distribution and a log link function, and including factors for both treatment and replicate.

## Results

### Identification of Candidate Pheromone Components

**Fig. 2:**
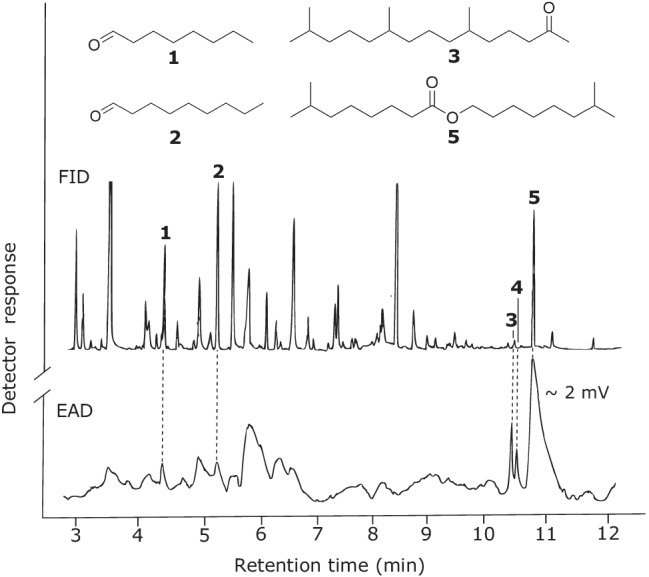
Representative responses of a gas chromatographic flame ionization detector (FID) and an electroantennographic detector (EAD: antenna of a male *Agriotes ferrugineipennis*) to aliquots of Porapak Q headspace volatile extract from conspecific females. Compounds **1**, **2**, **3**, and **5** were identified as octanal (**1**), nonanal (**2**), 6,10,14-trimethyl-2-pentadecanone (**3**), and 7-methyloctyl 7-methyloctanoate (**5**). Compound **4** was tentatively identified as 6,10,14-trimethyl-2-pentadecanol, but the amount present in the extract was not sufficient to obtain a mass spectrum for confirmation. Other apparent antennal responses could not be repeated on various GC columns. Chromatography: DB-5 column; temperature program: 50 °C for 1 min, then 20 °C · min^−1^ to 280 °C

**Fig. 3:**
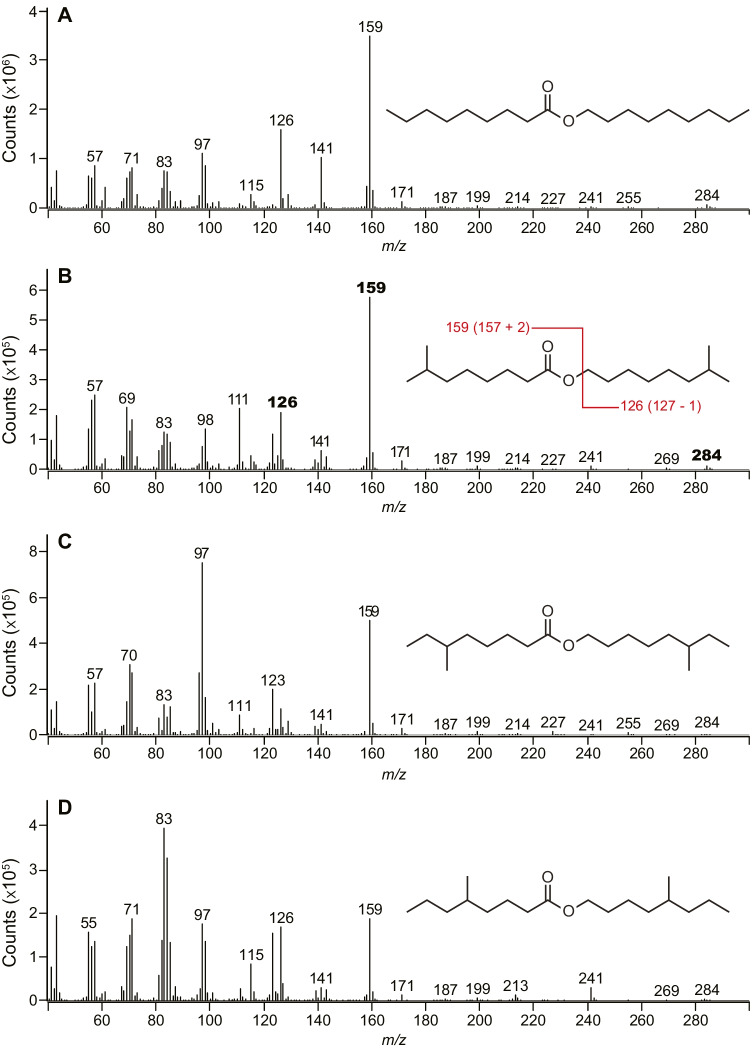
Mass spectra of synthetic nonyl nonanoate (**A**), 7-methyloctyl 7-methyloctanoate (7Me7Me) (**B**), 6-methyloctyl 6-methyloctanoate (**C**), and 5-methyloctyl 5-methyloctanoate (**D**) on a 5977A MSD (Agilent Technologies Inc.) coupled to a 7890B GC fitted with a DB-5MS column. The mass spectrum of 7Me7Me matched that of beetle-produced component **5** in Fig. 2

GC-EAD analyses of headspace volatile extracts of female *A. ferrugineipennis* revealed five components (**1**, **2**, **3, 4** and **5** in Fig. 2) that elicited responses from male *A. ferrugineipennis* antennae. Whereas some other FID peaks also appeared to elicit antennal responses, these responses could not be repeated in recordings on other GC columns. Unlike components **1**–**4**, component **5** was female-specific and elicited the strongest antennal responses. The mass spectrum of **5** showed a base peak (*m*/*z* 159) and a molecular ion (*m*/*z* 284) indicative of a nonyl nonanoate. Yet, synthetic nonyl nonanoate – prepared according to Neises and Steglich (1978) – had retention indices significantly higher than those of **5** on all four GC columns (Table 2), indicating that **5** had at least one methyl branch. With 7-methyloctyl nonanoate (available from a previous project) still eluting too late (Table 2), we considered octanoates with methyl branches in both the acid and alcohol part of the ester. Reviewing the literature for previously reported di-methyl octanoates in click beetles, we found a study by Tolasch et al. (2007) that reported the presence of 7-methyloctyl 7-methyloctanoate (7Me7Me) in pheromone gland extracts of female *Elater ferrugineus*. We synthesized 7Me7Me and determined that its mass spectrum (Fig. 3) and retention indices (Table 2) were entirely consistent with those of beetle-produced **5**. Moreover, beetle-produced and synthetic 7Me7Me, each tested at 10 ng, elicited comparably strong responses from male antennae in GC-EAD recordings. To unequivocally prove that the methyl branches of **5** were indeed at C-7, rather than at C-6 or C-5, we also synthesized 6-methyloctyl 6-methyloctanoate and 5-methyloctyl 5-methyloctanoate. As expected, neither the mass spectra nor the retention indices of these two esters matched those of beetle-produced **5** (Fig. 3, Table 2).

**Table 2:** Retention indices of straight-chain and methyl-branched esters on each of four GC columns. Note retention index matches between beetle-produced component **5** and synthetic 7-methyloctyl 7-methyloctanoate on each of four GC columns

	Retention indices			
Compounds	DB-5	DB-210	DB-23	FFAP
**Beetle-produced component 5** (in Fig. 2)	**1900**	**2184**	**2183**	**2128**
nonyl nonanoate	1975	2245	2278	2224
7-methyloctyl nonanoate	1937	2214	2230	2176
**7-methyloctyl 7-methyl octanoate**	**1900**	**2184**	**2183**	**2128**
6-methyloctyl 6-methyl octanoate	1917	2205	2215	2157
5-methyloctyl 5-methyl octanoate	1888	2149	2172	2116

GC–MS analyses of beetle-produced **1** and **2** confirmed that they were octanal (**1**) and nonanal (**2**). Beetle-produced **3**, with mass spectral fragmentation *m/z* 58 (indicative of a keto-group in C-2) and a molecular weight of 268 was identified as 6,10,14-trimethyl-2-pentadecanone by comparison with a synthetic standard at hand (Sasaerila et al. 2003). Beetle-produced **4** had the retention time of 6,10,14-trimethyl-2-pentadecanol (the corresponding alcohol of compound **3**) but the amount present in extracts was not sufficient to obtain a mass spectrum to confirm this tentative assignment.

### Field Experiment

**Fig. 4:**
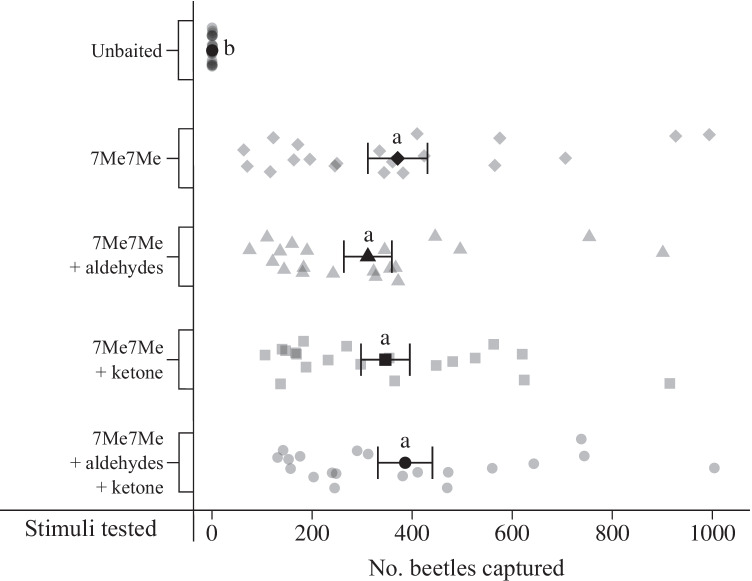
Captures of male click beetles, *Agriotes ferrugineipennis,* in a field experiment run near Pemberton (British Columbia) between 12 April and 03 May 2021 (*N* = 16 during weeks 1–3; *N* = 20 during weeks 4–7). Five treatments were tested: (1) unbaited (control); (2) 7-methyloctyl 7-methyloctanoate (7Me7Me) (10 mg); (3) 7Me7Me (10 mg) plus the aldehydes octanal (1 mg) and nonanal (1 mg); (4) 7Me7Me (10 mg) plus the ketone 6,10,14-trimethyl-2-pentadecanone (1 mg); and (5) 7Me7Me (10 mg) plus the two aldehydes (1 mg each) and the ketone (1 mg). Grey and black symbols show the number of beetles captured in each replicate and on average (mean ± standard error), respectively. Means with different letters indicate statistically significant differences in trap captures (generalized linear model fitted with a negative binomial distribution and a log link function; *P* < 0.05). Within each treatment, the data appear at slightly different heights due to a jitter function of the software program that is applied when the plot is produced

The proportion of *A. ferrugineipennis* males among all click beetles captured varied with both treatment (χ^2^ = 180.0, df = 4,137, *P* < 0.0001) and collection week (χ^2^ = 324.6, df = 6,137, *P* < 0.0001) (Table 1). Proportions of *A. ferrugineipennis* males were lowest in unbaited control traps, which captured mostly *A. obscurus*, *A. lineatus* (two invasive species recently found in the Pemberton area; van Herk et al. 2021c), and *Limonius canus*. There was no statistically significant difference (*P* > 0.05) in the proportion of *A. ferrugineipennis* males that were captured in traps baited with a 1-, 2-, 3- or 4-component blend of the CPCs (Fig. 4). The total number of *A. ferrugineipennis* males captured varied with both treatment (χ^2^ = 282.8, df = 4,76, *P* < 0.0001) and replicate (χ^2^ = 105.4, df = 19,76, *P* < 0.0001), with no statistically significant differences (*P* > 0.05) between CPC treatments, and with all captures in CPC-baited traps (range of means: 311.4–386.0) being significantly higher (*P* < 0.0001) than those in unbaited control traps (mean: 0.3) (Fig. 4). Small numbers of female *A. ferrugineipennis* (25)*,* and of male and female *A. lineatus* (63, 19), *A. obscurus* (20, 2), *L. canus* (172, 0), and unidentified elaterids (11, 9) were collected in both baited and non-baited traps.

## Discussion

Laboratory analyses and field trapping data indicate that 7-methyloctyl 7-methyloctanoate (7Me7Me) is the major sex pheromone component of female *A. ferrugineipennis.* The ester 7Me7Me elicited the strongest responses from male antennae in electrophysiological recordings, and all synthetic lures containing 7Me7Me in a field trapping experiment attracted large numbers of *A. ferrugineipennis* males. While octanal, nonanal and 6,10,14-trimethyl-2-pentadecanone are present in the headspace of female beetles, and are sensed by male antennae, these compounds do not enhance the attractiveness of 7Me7Me, at least not in the context as tested in our study.

Based on the abundance of 7Me7Me in the headspace of female *A. ferrugineipennis* and the strong responses it elicited from male antennae in GC-EAD recordings (Fig. 2), we hypothesized that 7Me7Me is the major sex pheromone component of female *A. ferrugineipennis.* We further hypothesized that its attractiveness may be enhanced by the minor candidate pheromone components that were less abundant and only modestly EAD-active. We designed our field experiment accordingly and baited traps with 7Me7Me alone, and in binary, ternary or quaternary combinations with the minor candidate pheromone components. All traps baited with 7Me7Me alone as a single lure constituent, or as part of a blend, captured – on average – nearly 1200-times more *A. ferrugineipennis* males than unbaited control traps (Fig. 4), supporting the conclusion that 7Me7Me is the major sex pheromone component of female *A. ferrugineipennis*.

Although octanal, nonanal and 6,10,14-trimethyl-2-pentadecanone are emitted by females and sensed by males (Fig. 2), they do not seem to play a role as synergistic sex attractant pheromone components (Fig. 4). Trap lures with or without these compounds were equally effective in attracting very large numbers of *A. ferrugineipennis* males. Conceivably, however, these compounds may express pheromonal activity when presented together with 7Me7Me at blend ratios wider, or narrower, than tested in our study. Alternatively, one or more of these compounds may have a pheromonal function in the context of species or mate recognition rather than mate attraction. If not, it would seem perplexing that *A. ferrugineipennis* females emit components which are both chemically diverse (ester, ketone, aldehydes) and recognized by males.

7-Methyloctyl 7-methyloctanoate, together with 7-methyloctyl 5-methylhexanoate, 7-methyloctyl octanoate and 7-methyloctyl (*Z*)-4-decenoate has previously been identified in pheromone gland extracts of female *Elater ferrugineus* (Tolasch et al. 2007), a rare predatory elaterid species inhabiting deciduous trees in Europe (Ranius et al. 2011). A synthetic blend of these four esters was field-tested and shown to attract *E. ferrugineus* males (Tolasch et al. 2007). A follow-up study (Svensson et al. 2012) then revealed that the pheromonal activity resides with 7-methyloctyl (*Z*)-4-decenoate as a single component. In electrophysiological recordings that tested the four esters, only 7-methyloctyl (*Z*)-4-decenoate elicited responses from male *E. ferrugineus* antennae, and in a field trapping experiment, only lures containing 7-methyloctyl (*Z*)-4-decenoate effectively attracted *E. ferrugineus* males, with the other three esters not contributing to the attractiveness of lures. While 7-methyloctyl 7-methyloctanoate (7Me7Me) has no pheromonal function in *E. ferrugineus*, it is the major sex attractant pheromone component of female *A. ferrugineipennis* (Figs. 2, 4) and is reported here as a new pheromone in the Insecta.

As 7Me7Me is produced by females of both *A. ferrugineipennis* (Elaterinae: Agriotini) and *E. ferrugineus* (Elaterninae: Elaterini), which represent two taxonomically distinct tribes with non-overlapping geographic distribution (Becker 1956; Tolasch et al. 2007; Nieto and Alexander 2010), it follows that the biosynthetic ability to produce 7Me7Me has evolved independently at least twice in the Elateridae, even though thus far it is a pheromone component only in *A. ferrugineipennis.*

The molecular structure of 7Me7Me differs from currently known *Agriotes* sex pheromones (Tóth 2013) which are commonly geranyl esters (Yatsynin and Rubanova, 1983; Yatsynin et al., 1980, 1991; Tóth et al., 2002, 2003; Siirde et al. 1993), farnesyl esters (Yatsynin et al. 1980, 1991; Tóth et al. 2003; Tolasch et al. 2022) and – rarely – neryl esters (Tolasch et al. 2010; Tolasch and Steidle 2022). Within the *Agriotes* genus*, A. ferrugineipennis* is placed in the *Limosus* group (Becker 1956) for which no sex pheromone was known prior to our study. It is conceivable that other species in the *Limosus* group produce sex pheromones similar to 7Me7Me but more species in this group must be studied before any generalization is warranted.

In conclusion, trap lures containing 7Me7Me were exceedingly attractive to mate-seeking males, suggesting that 7Me7Me may be the major, and possibly the only, sex attractant pheromone component of female *A. ferrugineipennis*. Octanal, nonanal and 6,10,14-trimethyl-2-pentadecanone in the headspace of *A. ferrugineipennis* are all sensed by males but do not seem to serve as (synergistic) sex attractant pheromone components. Our prediction that they function in the context of species or mate recognition, rather other mate attraction, will need to be tested in another study.

## Data Availability

All data are presented in the main body of the manuscript.
